# Amniotic fluid‐derived small extracellular vesicles for predicting postnatal severe outcome of congenital diaphragmatic hernia

**DOI:** 10.1002/jex2.160

**Published:** 2024-06-21

**Authors:** Seiko Matsuo, Akira Yokoi, Kosuke Yoshida, Masami Kitagawa, Eri Asano‐Inami, Mayo Miura, Takao Yasui, Sho Tano, Takafumi Ushida, Kenji Imai, Hiroaki Kajiyama, Tomomi Kotani

**Affiliations:** ^1^ Department of Obstetrics and Gynecology Nagoya University Graduate School of Medicine Nagoya Japan; ^2^ Nagoya University Institute for Advanced Research Nagoya Japan; ^3^ Japan Science and Technology Agency (JST) FOREST Kawaguchi Japan; ^4^ Department of Obstetrics and Gynecology Tokoname Municipal Hospital Tokoname Japan; ^5^ Department of Life Science and Technology Tokyo Institute of Technology Yokohama Japan; ^6^ Institute of Nano‐Life Systems, Institutes of Innovation for Future Society Nagoya University Nagoya Japan; ^7^ Department of Biomolecular Engineering, Graduate School of Engineering Nagoya University Nagoya Japan; ^8^ Division of Reproduction and Perinatology, Center for Maternal‐Neonatal Care Nagoya University Hospital Nagoya Japan

**Keywords:** amniotic fluid‐derived small extracellular vesicle, cellulose nanofiber sheet, congenital diaphragmatic hernia, microRNA, nitrofen‐induced rat model

## Abstract

Congenital diaphragmatic hernia (CDH) is a life‐threatening condition with high morbidity and mortality rates. The survival rate of neonates with severe CDH is reportedly only 10%–15%. However, prenatal prediction of severe cases is difficult, and the discovery of new predictive markers is an urgent issue. In this study, we focused on microRNAs (miRNAs) in amniotic fluid‐derived small EVs (AF‐sEVs). We identified four miRNAs (hsa‐miR‐127‐3p, hsa‐miR‐363‐3p, hsa‐miR‐493‐5p, and hsa‐miR‐615‐3p) with AUC > 0.8 to classify good prognosis group and poor prognosis group in human study. The AUC for hsa‐miR‐127‐3p and hsa‐miR‐615‐3p, for predicting the poor prognosis, were 0.93 and 0.91, respectively. In addition, in the in vivo study, the miRNA profiles of the lung tissues of CDH rats were different from those of control rats. Additionally, two elevated miRNAs (rno‐miR‐215‐5p and rno‐miR‐148a‐3p) in the lung tissues of CDH rats were increased in the AF‐sEVs of CDH rats. Our results suggest that severe CDH neonates can be predicted prenatally with high accuracy using miRNAs contained in AF‐sEVs. Furthermore, miRNA profile changes in AF‐sEVs reflected the lung status in CDH. Our findings may contribute to the development of advanced perinatal care for patients with CDH.

## 1 INTRODUCTION

Congenital diaphragmatic hernia (CDH) is a malformation that causes a defect in the diaphragm, with an incidence of 1 in 3000−4000 live births (Chatterjee et al., 2020; Keijzer & Puri, 2010; McGivern et al., 2015). It is characterized by high morbidity and mortality owing to pulmonary hypoplasia and hypertension caused by organ intrusion into the thoracic cavity (Wynn et al., 2013). In severe cases, the survival rate is only 10%−15% (Jani et al., 2007). Although foetal endotracheal occlusion (FETO) has improved the survival rate of severe cases from 24% to 49%, many patients still cannot be saved even with FETO treatment (Deprest et al., 2021). In addition, CDH foetuses not indicated for FETO can even result in neonatal death. Therefore, the development of new treatment strategies and prediction of disease severity are urgently required.

Predicting severe neonates is important for prenatal and postnatal treatment strategies and for counselling parents. The lung‐to‐head ratio (LHR), observed‐to‐expected (o/e) LHR, o/e total foetal lung volume, and liver herniation have been reported as prognostic factors; however, their predictive accuracy remains controversial (Akinkuotu et al., 2016; Brindle et al., 2014; Coughlin et al., 2016; Deprest et al., 2009; Lusk et al., 2015). The area under the curve (AUC) for survival prediction by these predictors was 0.70−0.91 (Jancelewicz & Brindle, 2020). o/e LHR is commonly used in clinical settings; however, inter‐observer variability and poor sensitivity are major problems (Bebbington et al., 2014). To identify novel biomarkers, we focused on small extracellular vesicles (sEVs) in amniotic fluids (AFs). EVs are lipid bilayer vesicles released from all living cells and contain proteins and nucleic acids such as microRNAs (miRNAs) (Colombo et al., 2014; Yokoi & Ochiya, 2021). They are stable in body fluids, and have gained increasing attention as disease biomarkers in various fields (Lu et al., 2019; Yokoi et al., 2018; Yu et al., 2021). In obstetrics, abnormal changes in EV concentration and composition have been reported in pregnancy‐related diseases, including preeclampsia, gestational diabetes mellitus, and preterm birth (Zhang et al., 2020). However, biomarkers derived from amniotic fluid‐derived small EVs (AF‐sEVs) have been poorly investigated. AFs, pregnancy‐specific body fluids, mainly consist of foetal renal secretions, but also contain pulmonary secretions (Cananzi & De Coppi, 2012). Therefore, AFs may reflect the foetal lung status, and clinically, lamellar body counts in AFs have been used as an indicator of foetal lung maturation (Mol et al., 1999). Therefore, we believe that injured lung‐derived EVs from CDH foetuses are reflected in AFs. miRNAs, which are small non‐coding RNAs 17−25 nucleotides in length, are the most well‐studied molecules in EVs (Liu et al., 2019). miRNAs play important roles in the post‐transcriptional regulation of biological processes. The expression profiles of miRNAs vary widely between normal and diseased tissues and exhibit condition‐specific characteristics. Increasing evidence has demonstrated that miRNAs can serve as biomarkers not only in tissues but also in EVs in body fluids (Etheridge et al., 2011; Liu et al., 2019).

Thus, we aimed to discover biomarkers for predicting severe CDH by focusing on miRNAs contained in AF‐sEVs. In addition, we assessed whether the miRNA profile changes in AF‐sEVs from CDH foetuses were caused by lung stress, using a rat model.

## 2 MATERIALS AND METHODS

Additional detailed methods are included in the Supplementary material.

### 2.1 Study population and sample collection

**FIGURE 1:**
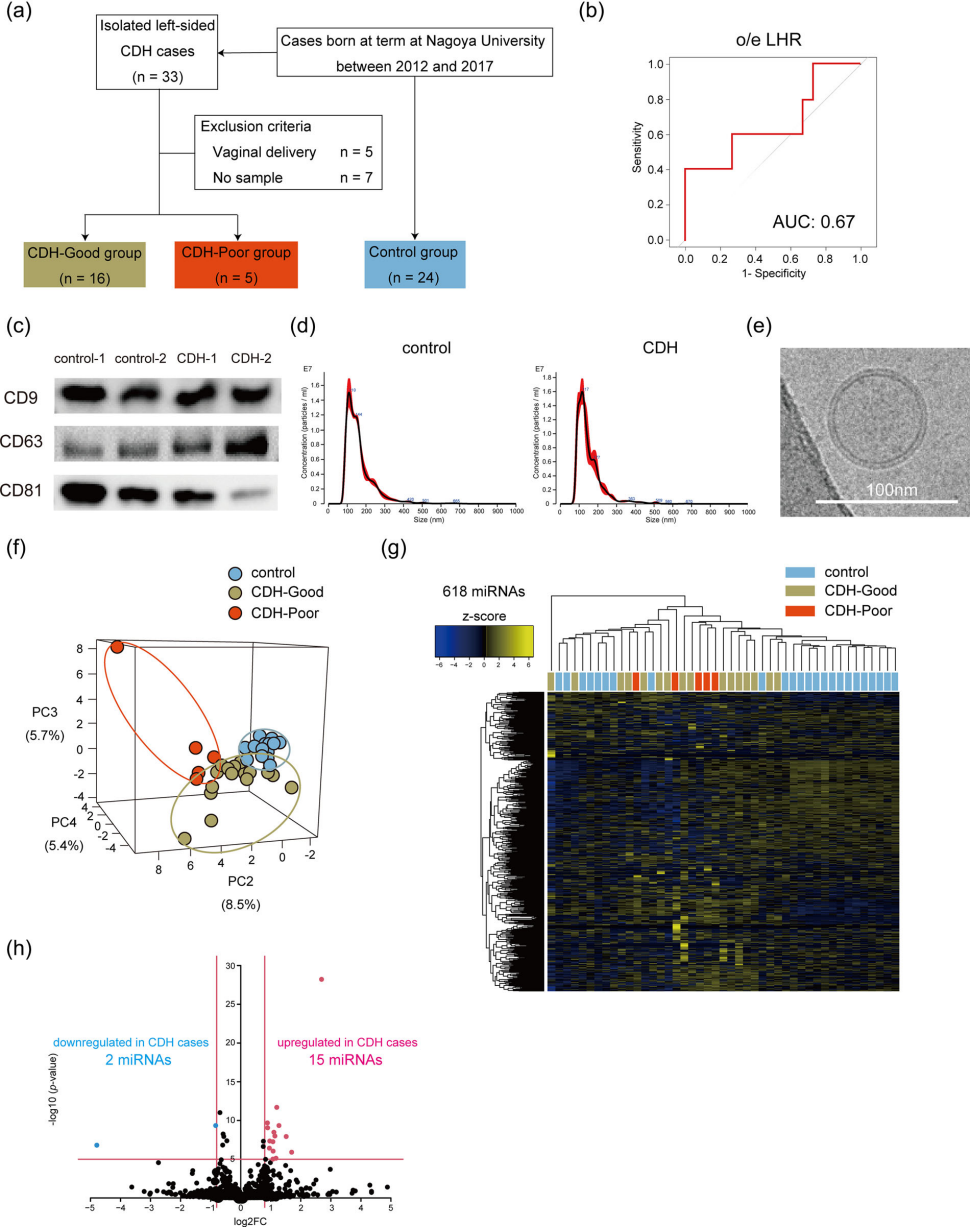
AF‐sEV isolation and small RNA sequencing. (a) Flow diagram of the study populations. Among the 33 isolated left‐sided CDH cases born at term, 21 cases were included in the analysis. CDH cases were divided into the good and poor prognosis groups. Neonates that required extracorporeal membrane oxygenation or neonatal deaths were defined as the CDH‐Poor (*n* = 5), and the others were defined as the CDH‐Good (*n* = 16). Twenty‐four matched control cases were also included in the analysis. (b) Receiver operating characteristic curves for predicting severe CDH cases using o/e LHR (continuous variables). (c) Tetraspanin (CD9, CD63 and CD81) marker expression in amniotic fluid‐derived small extracellular vesicles (AF‐sEVs). (d) Size distribution of AF‐sEVs from a control and CDH case was determined using nanoparticle tracking analysis. (e) Morphology of AF‐sEVs detected using transmission electron microscopy. Scale bar represents 100 nm. (f) Principal component analysis mapping for AF‐sEV miRNA expression of the control, CDH‐Good, and CDH‐Poor cases. Principal component plot using PCs with the best discrimination power (PC2–PC4) were shown. (g) Heatmap for AF‐sEV miRNA expression of the control, CDH‐Good and CDH‐Poor cases. (h) Volcano plot showing differentially expressed miRNAs between AF‐sEVs from control and CDH cases. CDH, congenital diaphragmatic hernia.

AF samples were collected during caesarean delivery from women who provided written informed consent between January 2012 and October 2017. Isolated left‐sided CDH neonates who were born at term (between 37^0/7^ and 41^6/7^ gestational weeks) and those who were born at term at the same time without congenital malformations or neonatal intensive care unit (NICU) admission (control cases: *n* = 24) were included (Figure 1a). Twenty‐one CDH cases were included in the analysis. CDH cases were divided into two groups: a good prognosis group (CDH‐Good; *n* = 16) and a poor prognosis group (CDH‐Poor; *n* = 5). Patients who required extracorporeal membrane oxygenation (ECMO) or died neonatally were defined as the CDH‐Poor group, while the others were defined as the CDH‐Good group. Blood contamination or meconium‐stained amniotic fluid was not observed. After centrifugation at 3000 rpm × *g* for 10 min at 4°C, the supernatant was collected and stored. The study protocol was approved by the Institutional Ethics Board of Nagoya University (approval number: 2022‐0038, approval date: May 11, 2022).

### 2.2 Isolation of EVs

The EV isolation method used in this study adhered to the standard principles of the International Society for Extracellular Vesicles (Théry et al., 2018; Witwer et al., 2013). Approximately 1 mL of each AF sample was centrifuged at 10,000 × *g* for 40 min at 4°C (Kubota 3520). The supernatant was filtered using a 0.22 μm filter (Millex‐GV 33 mm, Millipore), and then ultracentrifuged at 110,000 × *g* for 70 min at 4°C using a TLA55 rotor (Beckman Coulter Inc., USA). The pellet was washed with phosphate‐buffered saline (PBS), ultracentrifuged under the same conditions, and resuspended in PBS to extract the sEVs.

For the extraction of rat AF‐sEVs, we used cellulose nanofiber (CNF) sheets as we previously described (Yokoi et al., 2023). CNF sheets were attached to the surface of the rat foetus immediately after the incision of the amniotic membrane. After attaching for a few seconds, the sheets were dried. CNF sheets were dried for about 1‐week, then soaked in 1 mL of 0.22 μm filtered PBS, followed by vortex for 10 s and waiting for 5 min. The CNF sheets were removed and used as rat AF‐sEVs in subsequent experiments. The protein concentration of the EV fraction was determined using a Quant‐iT Protein Assay with a Qubit 2.0 Fluorometer (Invitrogen, Life Technologies, CA, USA).

### 2.3 Confirmation of EVs

To confirm successful sEV isolation and determine the size distribution of the sEVs, nanoparticle tracking analysis was performed using the Nanosight system (Nanosight, Quantum Design Inc., Japan) on samples diluted 50‐ to 100‐fold using PBS. The isolated sEVs were visualized using a phase‐contrast transmission electron microscope (Terabase Inc., Okazaki, Japan) to confirm the lipid bilayer structure. Immunoblot analysis was performed to determine the expression of exosome markers. Uncropped scans of the blots were shown in Figure S4.

To confirm the successful isolation of rat AF‐sEV, we used Nanosight (Quantum Design Inc) and transmission electron microscopy.

### 2.4 Animal model studies

The experimental procedures in this study were approved by the Animal Experiment Committee of Nagoya University Graduate School of Medicine (approval number: M220212‐002 in March 2022), complied with the ARRIVE guidelines, and were carried out according to the National Institutes of Health Guide for the Care and Use of Laboratory Animals. Pregnant Sprague‐Dawley rats were purchased from Japan SLC, Inc. (Hamamatsu, Japan). All rats were maintained on a 12 h light/12 h dark lighting schedule (lights on at 9:00 AM, off at 9:00 PM) and allowed ad libitum access to water and standard chow. A nitrofen‐induced rat CDH model was established as previously reported (Kluth et al., 1990; Miura et al., 2021). The protocol is shown in Figure 3a. Of the four pregnant rats, two were administered 1 mL olive oil (control) and two were administered 100 mg nitrofen (2,4‐dichlorophenyl‐p‐nitrophenylether, Sigma‐Aldrich, Inc., St. Louis, MO, USA) dissolved in 1 mL olive oil through a gastric tube on embryonic Day 9 (E9). On embryonic Day 21 (E21), the rats were euthanized with carbon dioxide and subjected to caesarean section. After the uterine incision, AF‐sEVs were extracted using CNF sheets. Foetuses were sacrificed by cervical dislocation, weighed, and checked for the presence of a diaphragm using thoracolaparotomy. Next, foetal lung tissues were collected in GentleMACS M tubes (Miltenyi Biotec, Bergisch Gladbach, Germany) containing 1.5 mL of QIAzol buffer (Qiagen, Hilden, Germany). The tissues were homogenized using the dissociation template RNA_01.01 program.

### 2.5 RNA extraction and small RNA sequencing

Total RNA was extracted from human AF‐sEVs and rat lungs using the miRNeasy Mini Kit (Qiagen), according to the manufacturer's protocols. For rat AF‐sEVs, RNA was extracted from CNF sheets using a Urine microRNA Purification Kit (Norgen Biotek Corporation, Ontario, Canada). The CNF sheets were soaked in 1.5 mL of lysis buffer A, vortexed for 15 s, and allowed to stand for 5 min. The CNF sheets were then removed, 99.5% ethanol was added, and it was vortexed for 10 s. The remaining procedures were performed according to the manufacturers’ instructions. Small RNA sequencing was performed using the MiSeq or the NextSeq instrument (Illumina, San Diego, CA, USA).

### 2.6 Statistical tests

RStudio (RStudio, Boston, MA) and R software (ver. 4.0.3) were used. For differentially expressed miRNAs in human AF‐sEVs, Dunnett's test was performed on the control, good, and poor cases, using the control as the reference. To determine the ability of the miRNAs to classify poor and good outcomes, receiver operating characteristic (ROC) curves were constructed. A *p‐value* < 0.05 was considered as statistically significant.

## 3 RESULTS

### 3.1 Patient characteristics, AF‐sEV characterization, and miRNA profiling

Twenty‐one neonates with CDH (CDH‐Good group, *n* = 16; CDH‐Poor group, *n* = 5) and 24 control cases were included in the analysis (Figure 1a). The patient characteristics are shown in Table 1. Gestational age was slightly shorter in CDH‐Poor group compared to the control group (37.4 ± 0.2, 38.1 ± 0.4, *p *< 0.05). In our population, only two CDH‐Poor neonates could be predicted using the o/e LHR. The AUC for prediction of severe neonates in this study population using o/e LHR (continuous variables) and o/e LHR < 25 was 0.67 and 0.70, respectively (Figure 1b, Figure S1).

**TABLE 1:** Patient characteristics.

	Control (*n* = 24)	CDH‐Good (*n* = 16)	CDH‐Poor (*n* = 5)	*p* value
Gestational age at delivery (week)	38.1 ± 0.4	38.0 ± 0.9	37.4 ± 0.2	<0.05
Birth weight (g)	2964 ± 254	2840 ± 328	2783 ± 288	0.27
Male	14/24 (58.3%)	9/16 (56.3%)	3/5 (60.0%)	1.00
o/e LHR (%)	n/a	44.2 ± 17.6	33.6 ± 22.0	0.28
o/e LHR < 25%	n/a	0/15 (0%)	2/5 (40%)	0.05

*Note*: Data are shown as mean ± standard deviation or *n* (%). Tested using one‐way analysis of variance.

Abbreviations: CDH, congenital diaphragmatic hernia; o/e LHR, observed‐to‐expected lung‐to‐head ratio.

AF‐sEVs were isolated by ultracentrifugation. Western blot analysis confirmed the expression of EV‐specific markers (CD9, CD63 and CD81) and the absence of GRP94 (Figure 1c, Figure S4d). In nanoparticle tracking analysis, the control and CDH AF‐sEVs showed similar size distributions (Figure 1d). Mean particle size was 164.5 ± 2.8 nm and 153.6 ± 1.0 nm for control and CDH AF‐sEVs, respectively. The morphology of the control AF‐sEVs was confirmed by cryoelectron microscopy (Figure 1e). Based on these data, we confirmed that EVs were successfully isolated from AFs.

We extracted RNAs from the AF‐sEVs and performed small RNA sequencing. Principal component analysis (PCA) and heatmap revealed that the miRNA profiles of AF‐sEVs in the control, CDH‐Good, and CDH‐Poor groups were significantly different (Figures 1f and g). Principal components plot with the maximum variances (PC1–PC3) are shown in Figure S2.

### 3.2 miRNA candidate selection and predictive performance of differentially expressed miRNAs

**FIGURE 2:**
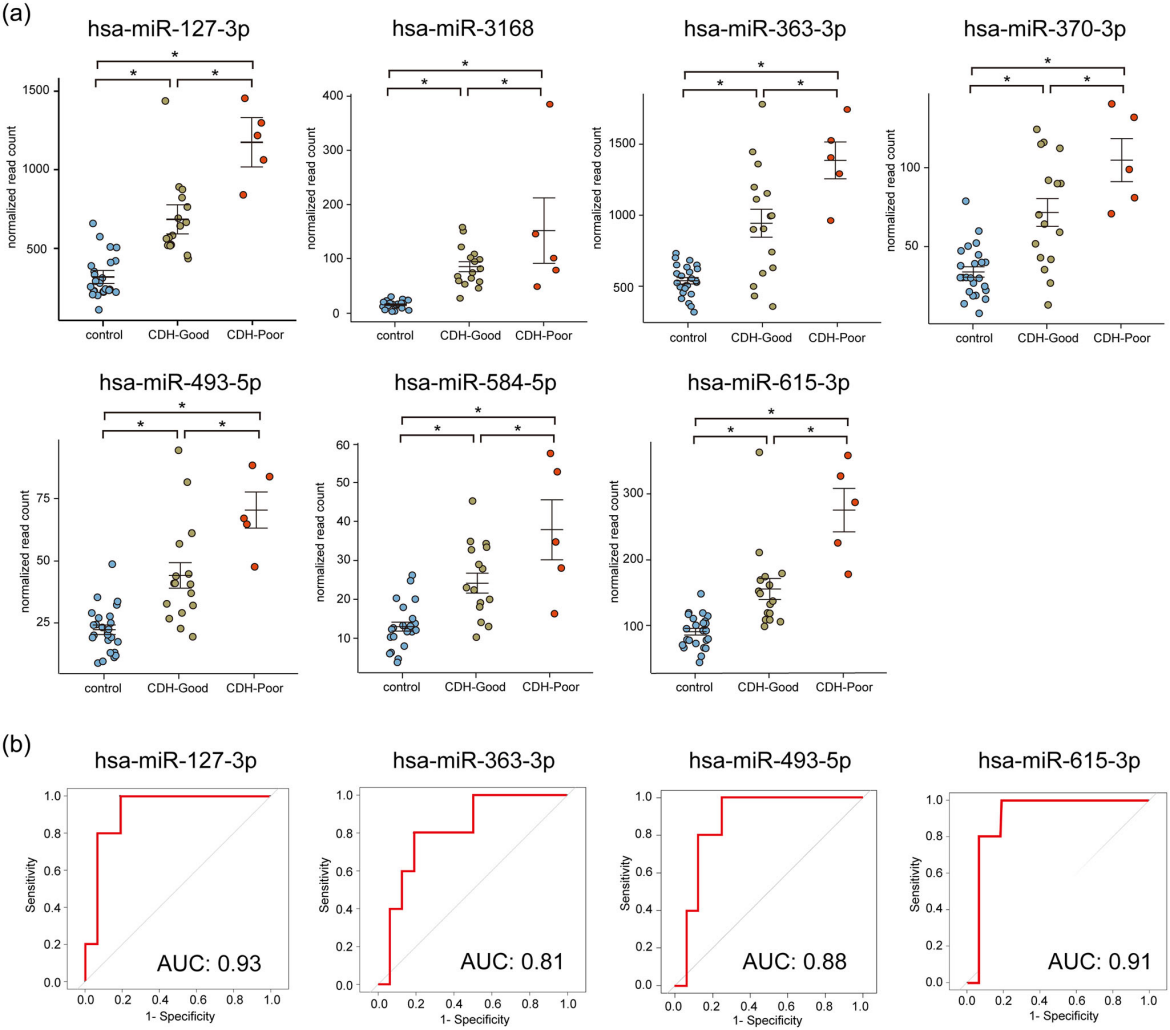
Selection of miRNA candidates and their predictive performance. (a) Dot plot of normalized read count of each miRNA in amniotic fluid‐derived small extracellular vesicles from the control, CDH‐Good and CDH‐Poor cases. Among the differentially expressed miRNAs, those with significant differences in CDH‐Good and CDH‐Poor (*p *< 0.05) and linear expression change were selected. The Dunnett's test was performed on the control, CDH‐Good, and CDH‐poor groups, using the control as the reference. *p < 0.05. (b) Receiver operating characteristic curves of four miRNAs for detecting CDH‐Poor cases from CDH‐Good cases. AUC was calculated and miRNAs with AUC > 0.8 were selected. AUC, area under the curve; CDH, congenital diaphragmatic hernia.

Differentially expressed miRNAs were selected by volcano‐plot filtering. Volcano plots showed that 15 miRNAs were significantly upregulated, whereas two miRNAs were significantly downregulated in AF‐sEVs from CDH cases compared to control cases (Figure 1h). Differentially expressed miRNAs are listed in Table S1. Among the 17 differentially expressed miRNAs, those with significant differences in the CDH‐Good and CDH‐Poor groups (*p *< 0.05) and linear expression change (control < CDH‐Good < CDH‐Poor or control > CDH‐Good > CDH‐Poor) were selected for further analysis (hsa‐miR‐127‐3p, hsa‐miR‐3168, hsa‐miR‐363‐3p, hsa‐miR‐370‐3p, hsa‐miR‐493‐5p, hsa‐miR‐584‐5p and hsa‐miR‐615‐3p). A dot plot of the normalized read count of the selected miRNAs is shown in Figure 2a, and the others are shown in Figure S3. Next, to determine the ability of miRNAs to classify the CDH‐Good and CDH‐Poor groups, ROC curves were constructed for the seven miRNA candidates and miRNAs with an AUC > 0.8 were selected and ROC curves are shown in Figure 2b. Four miRNAs were identified. Each miRNA had high predictive performance, with hsa‐miR‐127‐3p and hsa‐miR‐615‐3p having AUC > 0.9. The AUC values for the unselected miRNAs (hsa‐miR‐3168, hsa‐miR‐370‐3p and hsa‐miR‐584‐5p) were 0.64, 0.78 and 0.75, respectively.

### 3.3 Animal model studies

**FIGURE 3:**
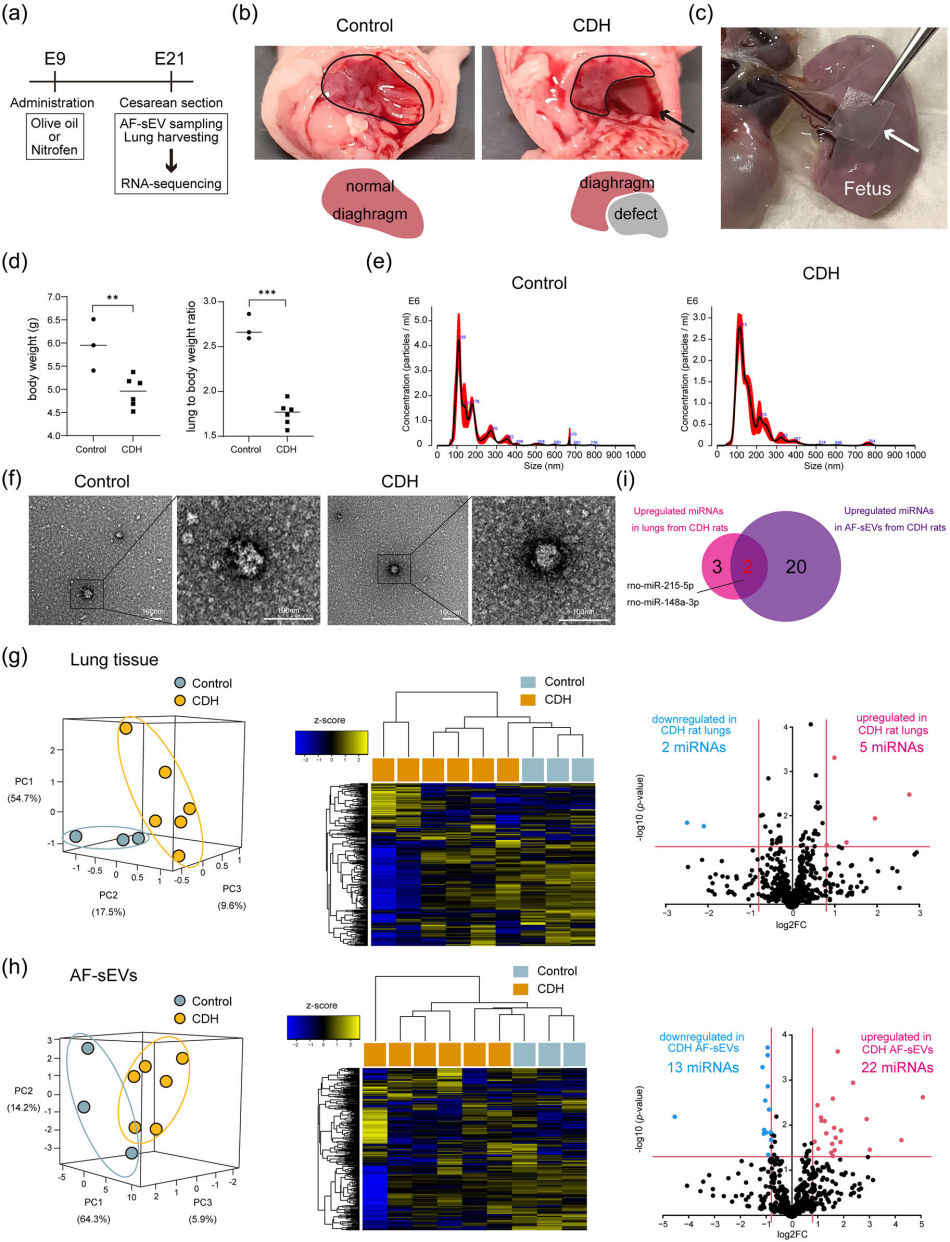
CDH rat model establishment and small RNA sequencing of lung tissue and amniotic fluid‐derived small extracellular vesicles (AF‐sEVs). (a) Experimental scheme of in vivo studies. For establishing CDH rat model, 100 mg nitrofen was orally administered on embryonic Day 9. As for control, pregnant rats were administered 1 mL olive oil instead of nitrofen. A caesarean section was performed on embryonic Day 21 to check for CDH and lungs and AF‐sEVs were harvested. (b) Photographs of the diaphragm of the control and CDH rats. CDH rats showed a large defect in the diaphragm. Schematic diagrams are shown below the photos. (c) Photograph of the rat AF‐sEV collection using the CNF sheet. White arrow indicates CNF sheet. (d) The body weight and the lung‐to‐body weight ratio of control and CDH rat foetuses. **p < 0.01, ***p < 0.001, Student's *t*‐test. (e) Size distribution of AF‐sEVs from a control and CDH rat foetus was determined using nanoparticle tracking analysis. (F) Morphology of AF‐sEVs from control and CDH rats isolated using CNF sheets detected by transmission electron microscopy. Scale bar represents 100 nm. (g) Expression analysis of miRNAs obtained from lung tissue of rats. PCA mapping and heatmap for miRNA expression of control and CDH rat lungs. Blue and orange show control and CDH rats, respectively. Volcano plot showing differentially expressed miRNAs between lungs from the control and CDH rats. (h) Expression analysis of miRNAs from AF‐sEVs of rat foetuses. PCA mapping and heatmap for miRNA expression of control and CDH rat AF‐sEVs. Blue and orange show control and CDH rats, respectively. Volcano plot showing differentially expressed miRNAs between AF‐sEVs from the control and CDH rats. (i) Venn diagram showing upregulated miRNAs in CDH lung tissue and AF‐sEVs compared to control. Two overlapped miRNAs existed. CDH, congenital diaphragmatic hernia; PCA, principal component analysis.

Eight of the 26 (30.8%) rat foetuses from nitrofen‐administered rats showed a defect in the diaphragm, diagnosed as CDH (Figure 3b). As shown in Figure 3d, the body weight and lung‐to‐body weight ratio of the rat‐CDH group were significantly lower than those of the rat‐control group. On embryonic Day 21, AF‐sEVs were collected from the foetal surface using CNF sheets, and lungs were harvested from both the rat‐CDH and ‐control groups (Figure 3c). We confirmed the successful AF‐sEV isolation using nanoparticle tracking analysis (Figure 3e). Mean particle size from AF‐sEVs was 174.4 ± 3.8 and 171.9 ± 7.0 nm for the rat‐control and ‐CDH groups, respectively. The morphology of control and CDH rat AF‐sEVs was confirmed by transmission electron microscopy (Figure 3f).

Next, we extracted RNAs from the lung tissues and AF‐sEVs and performed small RNA sequencing. PCA and heatmap showed that miRNA profiles were different in the rat‐CDH group compared to the rat‐control group, both in lung tissues (Figure 3g) and AF‐sEVs (Figure 3h). The volcano plot revealed that five miRNAs were significantly upregulated in the CDH lungs compared to the control lungs (Figure 3g) and that 22 miRNAs were significantly upregulated in the CDH AF‐sEVs compared to the control AF‐sEVs (Figure 3h). The differentially expressed miRNAs in the lungs and AF‐sEVs are shown in Tables S2 and S3, respectively. The Venn diagram shows that among the five miRNAs upregulated in CDH rat lung tissues, two (rno‐miR‐215‐5p and rno‐miR‐148a‐3p) were also elevated in CDH AF‐sEVs (Figure 3i).

## 4 DISCUSSION

The main finding of this study is that miRNAs contained in AF‐sEVs could be a best biomarker for detecting severe neonates with CDH prenatally. Furthermore, in vivo experiments demonstrate that miRNA profile changes in AF‐sEVs reflected the lung condition of CDH.

In previous reports, AUC for predicting severe CDH using ultrasound and magnetic resonance imaging ranged from 0.70 to 0.91 (Jancelewicz & Brindle, 2020). o/e LHR, which is used to determine FETO indications, is reported to have an AUC of 0.80; however, poor sensitivity and high inter‐observer variability are its weaknesses (Alfaraj et al., 2011; Bebbington et al., 2014). In this study, the AUC for predicting severe neonates with CDH using o/e LHR was up to 0.70. Therefore, molecular biomarker associated with poor prognosis have been poorly investigated. In the present prediction method using miRNAs present in AF‐sEVs, the AUC of hsa‐miR‐127‐3p and hsa‐miR‐615‐3p were 0.93 and 0.91, respectively, suggesting that these biomarkers may be able to predict severe CDH with higher accuracy than conventional prediction methods.

We identified AF‐sEV miRNA candidates that were upregulated in CDH compared to the controls and further upregulated in the CDH‐Poor group compared to the CDH‐Good group. Therefore, we hypothesized that miRNA candidates might be derived from the lungs of severe CDH cases compressed from intra‐abdominal organs. Among the four miRNA candidates, the overexpression of miR‐127 in rat foetal lung organ cultures resulted in impaired lung development (Bhaskaran et al., 2009). miR‐363‐3p upregulated in the lungs of nitrofen‐induced CDH rat models (Zhu et al., 2016) and inhibited rat lung alveolar type II cell proliferation (Zheng et al., 2021). The remaining two miRNAs have not been reported to be associated with lungs. According to a study characterizing AF‐EVs, the term AF‐EVs represents a heterogeneous population of EVs from multiple organs, including the placenta, foetal urine, and stem cells (Gebara et al., 2022). Although there are methods for predicting the origin of EVs using surface markers, methods for identifying lung‐derived EVs remain to be developed. Therefore, in this study, we used a rat CDH model to confirm our hypothesis that the miRNAs upregulated in CDH AF‐sEV originate from the compressed lungs. Two of the five miRNAs upregulated in the lungs of CDH rats, rno‐miR‐215‐5p and rno‐miR‐148a‐3p, were also upregulated in the AF‐sEVs of CDH rats. Thus, the miRNAs upregulated in the lungs of CDH mice also altered the miRNA profile of AF‐sEVs. miR‐215‐5p and miR‐148a‐3p were not significantly different between human CDH‐Good and CDH‐Poor AF‐sEVs. The lack of common miRNAs in humans and rats may be due to the different pathogeneses of human CDH and nitrofen‐induced rat models, and the fact that the severity of CDH was not taken into account in the rats. In addition, although human and rat miRNAs are highly homologous, their functions in rats remain unclear. To the best of our knowledge, there are no reports on the functions of rno‐miR‐215‐5p and rno‐miR‐148a‐3p in rat lungs. Further studies are required to confirm these findings.

To date, one study has reported differentially expressed miRNAs in AF‐sEVs of non‐survivors compared with survivors of CDH cases (Fabietti et al., 2021). However, real‐time PCR for a limited number of miRNAs was performed in this study instead of global sequencing. In addition, only CDH cases in which FETO was performed were included in the analysis, and there were no comparisons with control cases without CDH. Furthermore, this study did not discuss the prediction of non‐survivors using miRNAs. To our knowledge, this is the first study to predict severe CDH using miRNAs in AF‐sEVs.

Accurate prenatal prediction of severe neonates of CDH may allow for the following: (1) FETO can be performed on foetuses with an o/e LHR ≥ 25 but predicted to be severe based on EV‐miRNA analysis. (2) Early anticipated transition to ECMO. (3) Appropriate delivery planning and prenatal counselling for parents. We believe that the ideal timing for AF collection is when genetic amniocentesis, amnioreduction, or FETO is performed. A prospective multicentre validation study using prenatal AFs would be required to confirm the utility of miRNAs in AF‐sEVs.

This study has several strengths. First, we compared EV‐miRNAs in control and CDH AFs to narrow down the candidates because the number of CDH cases was not large, and we identified miRNAs with high prediction accuracy. Second, although it was difficult to isolate lung‐derived EVs, we used a CDH rat model and found an overlap between miRNAs upregulated in CDH lungs compared to control lungs and those upregulated in CDH AF‐sEVs compared to control AF‐sEVs, indicating that changes in miRNAs derived from CDH lungs were reflected in AF‐sEVs. Third, we used a novel technique using CNF sheets to collect rat AF‐sEVs, because AFs from rats, especially nitrofen‐induced CDH rats, are too scarce to collect sufficient amounts for ultracentrifugation. We confirmed that the rat AF‐sEVs collected using CNF sheets were successfully isolated. Finally, to the best of our knowledge, this is the first study in which rat AF‐sEVs from individual foetuses were isolated and compared. There were also several limitations of our study. First, the number of CDH cases was relatively small. However, high prediction accuracy was achieved by also analysing the controls. Second, validation in other cohorts was not possible due to the rarity of this disease. Third, the present study used the term AFs collected during caesarean sections, although prenatal AFs are suitable for prediction.

In conclusion, severe CDH cases can be prenatally predicted with high accuracy using miRNAs present in AF‐sEVs. Furthermore, miRNA profile changes in AF‐sEVs reflected the lung status in CDH. We believe that the prenatal prediction of severe CDH can lead to expanded indications for foetal treatment, optimization of postnatal treatment, and better prenatal counselling for parents. Further research is required to confirm these findings.

## GEOLOCATION INFORMATION

Department of Obstetrics and Gynecology, Nagoya University Graduate School of Medicine, Nagoya, Japan (35.157778N, 136.921667E).

## AUTHOR CONTRIBUTIONS

**Seiko Matsuo**: Conceptualization. **Akira Yokoi and Tomomi Kotani**: Conceptualization. **Akira Yokoi**: Methodology. **Kosuke Yoshida**: Methodology. **Masami Kitagawa**: Methodology. **Eri Asano‐Inami**: Methodology. **Mayo Miura and Takao Yasui**: Methodology. **Masami Kitagawa**: Investigation. **Mayo Miura**: Investigation. **Sho Tano**: Investigation. **Takafumi Ushida**: Investigation. **Kenji Imai and Tomomi Kotani**: Investigation. **Akira Yokoi**: Funding acquisition. **Akira Yokoi and Tomomi Kotani**: Project administration. **Akira Yokoi**: Supervision. **Takao Yasui**: Supervision. **Hiroaki Kajiyama and Tomomi Kotani**: Supervision. **Seiko Matsuo and Akira Yokoi**: Writing—original draft. **Seiko Matsuo**: Writing—review and editing. **Akira Yokoi**: Writing—review and editing. **Kosuke Yoshida and Tomomi Kotani**: Writing—review and editing. All the authors discussed the results and commented on the manuscript.

## CONFLICT OF INTEREST STATEMENT

The authors declare no conflicts of interest.

## HUMAN RIGHTS STATEMENTS AND INFORMED CONSENT

The study was approved by the Institutional Ethics Board of Nagoya University (approval number: 2022‐0038). Pre‐existing samples and medical records were used. Thus, we provided disclosure information on the methods of this study and gave the subjects opportunities to reject enrol in this study.

## Supporting information

Supporting Information

Supporting Information

Supporting Information

Supporting Information

## Data Availability

Small RNA sequence data were deposited in the Gene Expression Omnibus (GEO) under the accession numbers GSE240554 and GSE240558.
