# Supplementary material for: Amniotic fluid‐derived small extracellular vesicles for predicting postnatal severe outcome of congenital diaphragmatic hernia
Source: J Extracell Biol. 2024 Jun 21;3(6):e160. doi: 10.1002/jex2.160 (PMC11212330; doi:10.1002/jex2.160)
Supplement: Supplementary file 1 — Supporting Information [file JEX2-3-e160-s002.docx]

**Additional detailed methods**

**Immunoblot analysis**

EV samples containing 2.5 μg of protein were denatured at 95 ℃ in sample buffer solution with 3-Mercapto-1,2-propanediol (FUJIFILM Wako Pure Chemical Corporation, Osaka, Japan) for 5 min and then loaded onto polyacrylamide gels for electrophoretic separation of proteins at 30 mA. Proteins were transferred onto polyvinylidene difluoride membranes. After blocking with Blocking One (Nacalai Tesque Inc., Japan) for 1 h at 15–25 ℃, the membranes were incubated overnight at 4 ℃ with the following primary antibodies: mouse monoclonal anti-CD9 (CBL162, Merck), rabbit monoclonal anti-CD63 (EXOAB-CD63A-1; System Biosciences, LLC, CA, USA), mouse monoclonal anti-CD81 (sc-166029; Santa Cruz Biotechnology, TX, USA), and mouse monoclonal anti-GRP94 (sc-393402; Santa Cruz Biotechnology, TX, USA). Subsequently, the membranes were washed thrice for 5 min using Tris-buffered saline with 0.1% Tween® 20, then incubated for 1–3 h at 15–25 ℃ with secondary horseradish peroxidase-conjugated mouse anti-rabbit IgG (NA934-1ML; Cytiva Lifesciences, USA; dilution 1:5,000) or anti-mouse IgG (NA931-1ML; Cytiva; dilution 1:2,000) antibodies. Membranes were imaged using an ImageQuant LAS 4010 (GE Healthcare, IL, USA).

**Transmission electron microscopy for rat AF-sEVs**

Transmission electron microscopy of rat AF-sEVs was performed as follows. In brief, 10 µL EVs solubilized in ultrapure water were dropped on to Parafilm (Bemis Company, Inc., Neenah, WI, USA). A carbon-coated Formvar copper grid (catalog no. 645, Nisshin EM Co., Japan) was placed on the droplet to immerse its coated side and incubated for 30 s at 15–25 ℃. After negative staining with a 2% uranium diacetate solution for 1 min, the excess solution was dabbed with a piece of filter paper, and the samples were dried at 15–25 ℃. The grid was visualized at increasing magnifications up to 50 K using a JEM-2100 high-resolution transmission electron microscope (JEOL Co., Japan).

**Small RNA sequencing**

Small RNA libraries were prepared using the NEBNext Multiplex Small RNA Library Prep Set for Illumina (New England Biolabs, Ipswich, MA, USA), and index codes were added to attribute the sequences to each sample. Next, the polymerase chain reaction (PCR) products were purified using a QIAquick PCR Purification Kit (Qiagen) and a 6% TBE gel (120 V, 60 min). Furthermore, DNA fragments corresponding to 140–160 bp (the length of small non-coding RNA plus the 3′ and 5′ adaptors) were recovered, and the complementary DNAs concentration was measured using the Qubit dsDNA HS Assay Kit and a Qubit2.0 Fluorometer (Life Technologies, Carlsbad, CA). Finally, single-end reads were generated using an Illumina MiSeq or NextSeq (Illumina, San Diego, CA, USA).

The raw data files of small RNA sequencing were analyzed using the CLC Genomics Workbench version 9.5.3 (Qiagen). After adaptor trimming, the data were mapped to the miRbase 22 database, allowing up to two mismatches, and normalized using reads per million (RPM) mapped reads. For human EV-miRNA analysis, after excluding miRNAs with RPM max < 10 and one case with a markedly different expression pattern, 618 miRNAs were selected for subsequent analyses. For rat miRNA analysis, after excluding miRNAs with an RPM max < 100, 269 and 299 miRNAs were selected for subsequent analysis of the lungs and AF-sEVs, respectively. The heatmap.2 function of the gplot package (ver. 3.1.0) was used for heatmap and hierarchical clustering analyses. To visualize the volcano plots, the log2 fold change (log2FC) and adjusted *p*-values for each gene were calculated using the Wald test in DESeq2 (ver. 1.30.0). Differentially expressed miRNAs in human AF-sEVs of CDH cases compared to control cases were selected with an adjusted *p*-value < 0.00001 and an absolute log2FC > 0.8 as the cutoff criterion. Differentially expressed miRNAs in the lung tissue and AF-sEVs of CDH rats compared to controls were selected with an adjusted *p*-value < 0.05, and an absolute log2FC > 0.8 as the cutoff criterion.

**Supplementary Figures**

**Supplementary Fig. S1. Receiver operating characteristic curves for predicting severe neonates with congenital hernia using o/e LHR < 25%.**


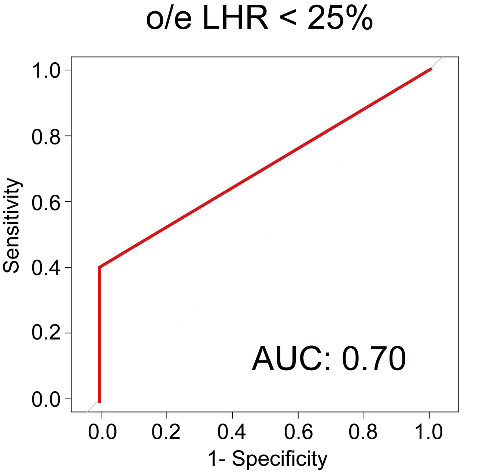


Area under the curve was calculated.

**Supplementary Fig. S2. Principal component mapping for AF-sEV miRNA expression of the control, CDH-Good, and CDH-Poor cases.**


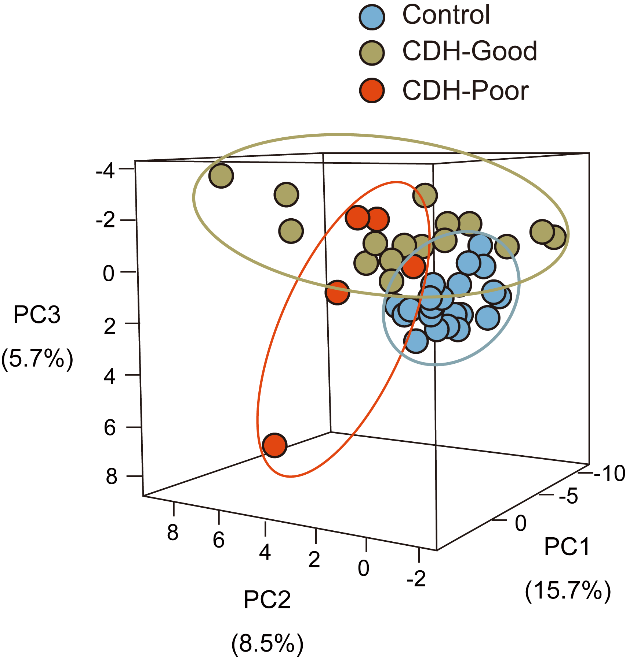


Principal component plot using principal components (PCs) with the maximum variants (PC1–PC3) were shown.

**Supplementary Fig. S3. Dot plot of normalized read count of unselected miRNAs.**


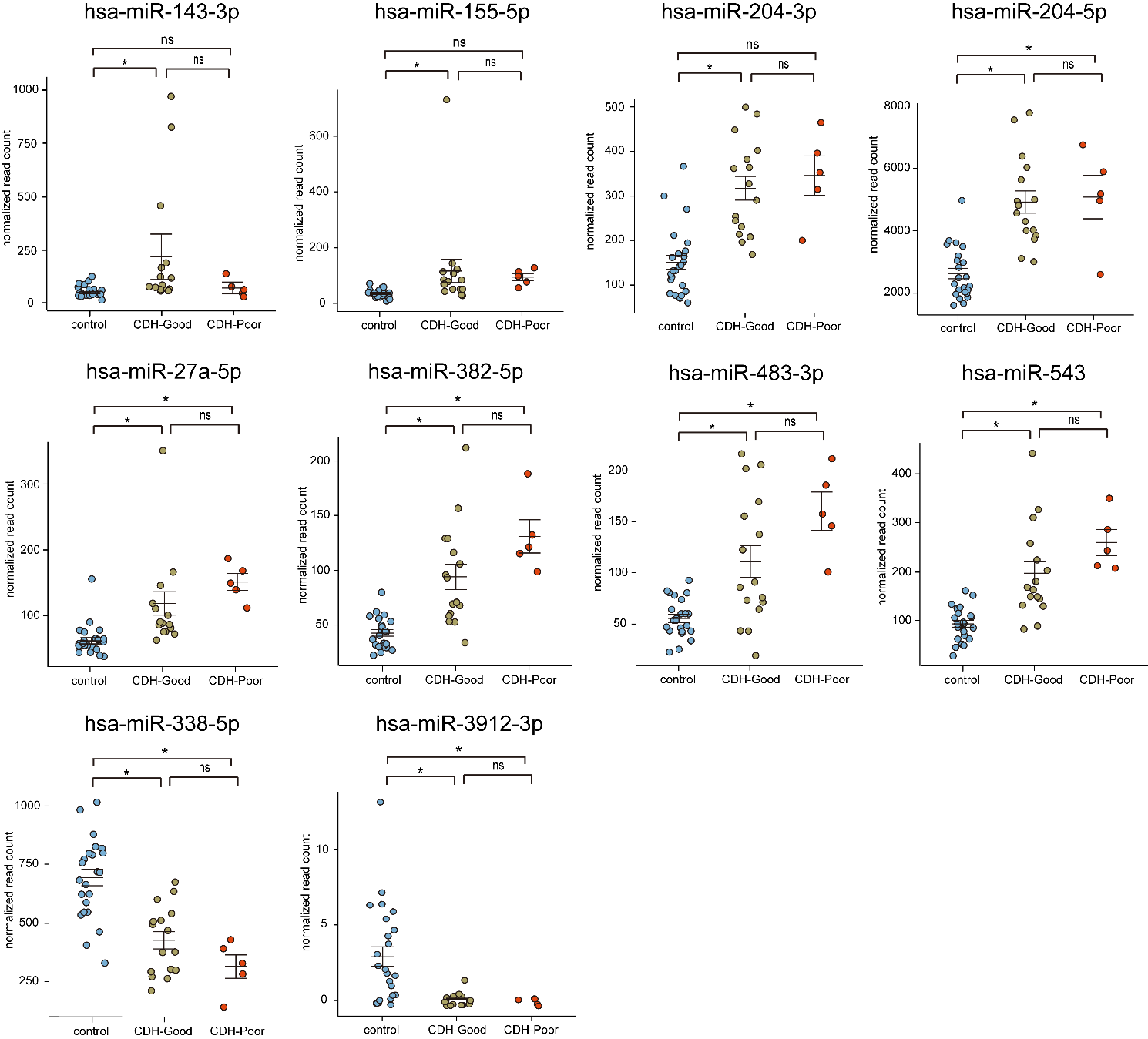


Dot plots of normalized read counts of each miRNA in AF-sEVs from the control, congenital diaphragmatic hernia (CDH)-Good, and CDH-Poor groups. Unselected miRNAs from 17 differentially expressed miRNAs in AF-sEVs of the control and CDH groups are shown. Dunnett’s test was performed on the control, CDH-Good, and CDH-Poor groups using the control as a reference. **P* < 0.05.

**Supplementary Fig. S4. Uncropped blot for western blot analysis.**


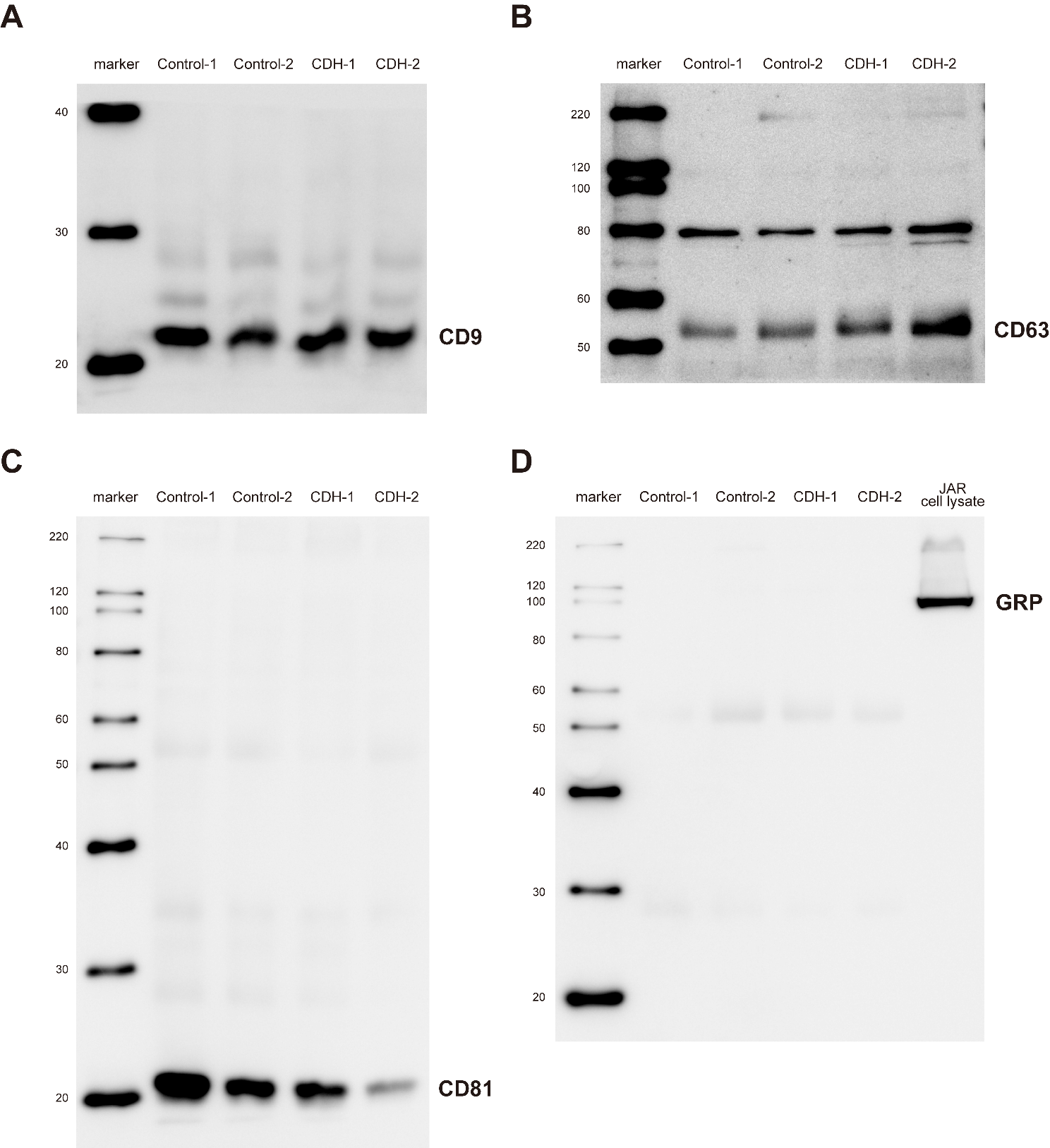


(A–C) Uncropped blot for Fig. 1C.

(D) Uncropped blots for western blot analysis of GRP94 expression. For GRP94 positive control, JAR choriocarcinoma cell line was used. JAR was obtained from the American Type Culture Collection. JAR was cultured in Dulbecco’s modified Eagle’s medium (4500 mg/L glucose) (Nacalai Tesque) supplemented with 10% fetal bovine serum and 1% penicillin-streptomycin. Confluent JAR cells in 6-well plate were rinsed with phosphate buffered saline and were collected in 200 µL of radioimmunoprecipitation assay buffer and the protein concentration was determined using the bicinchoninic acid assay (FUJIFILM Wako Pure Chemical Co., Japan).
